# Effect of Adjuvant Chemotherapy on Localized Malignant Giant Cell Tumor of Bone: A Systematic Review

**DOI:** 10.3390/cancers13215410

**Published:** 2021-10-28

**Authors:** Rokuro Morii, Shinji Tsukamoto, Alberto Righi, Kanya Honoki, Yuu Tanaka, Akira Kido, Hiromasa Fujii, Andreas F. Mavrogenis, Yasuhito Tanaka, Costantino Errani

**Affiliations:** 1 Department of Orthopaedic Surgery, Nara Medical University, 840, Shijo-cho, Kashihara-City 634-8521, Nara, Japan; K135367@naramed-u.ac.jp (R.M.); kahonoki@naramed-u.ac.jp (K.H.); hiro061211@yahoo.co.jp (H.F.); yatanaka@naramed-u.ac.jp (Y.T.); 2 Department of Pathology, IRCCS Istituto Ortopedico Rizzoli, Via Pupilli 1, 40136 Bologna, Italy; alberto.righi@ior.it; 3 Department of Rehabilitation Medicine, Wakayama Professional University of Rehabilitation, 3-1, Minamoto-Cho, Wakayama-City 640-8222, Wakayama, Japan; tanakayuu717@gmail.com; 4 Department of Rehabilitation Medicine, Nara Medical University, 840, Shijo-cho, Kashihara-City 634-8521, Nara, Japan; akirakid@naramed-u.ac.jp; 5 First Department of Orthopaedics, School of Medicine, National and Kapodistrian University of Athens, 41 Ventouri Street, Holargos, 15562 Athens, Greece; afm@otenet.gr; 6 Department of Orthopaedic Oncology, IRCCS Istituto Ortopedico Rizzoli, Via Pupilli 1, 40136 Bologna, Italy; costantino.errani@ior.it

**Keywords:** giant cell tumor of bone, localized, malignant, adjuvant, chemotherapy, surgery, prognosis

## Abstract

**Simple Summary:**

The effect of adjuvant chemotherapy on localized malignant giant cell tumors of the bone (GCTB) is unclear. We compared the mortality associated with wide resection compared to wide resection plus adjuvant chemotherapy for localized primary and secondary localized malignant GCTB. Among 745 relevant studies, 9 were included, with 39 and 73 primary and secondary malignant patients. In primary localized malignant GCTB, the mortality rates were 40% (6/15 patients) and 33% (8/24 patients) in the surgery plus adjuvant chemotherapy and surgery-only groups, respectively. The overall pooled odds ratio was 1.07 (*p* = 0.92). In secondary localized malignant GCTB, the mortality rates were 30.6% (11/36 patients) and 62.2% (23/37 patients) in the surgery plus adjuvant chemotherapy and surgery-only groups, respectively. The overall pooled odds ratio was 0.31 (*p* = 0.04). The effect of adjuvant chemotherapy remains unclear for primary localized malignant GCTB, but adjuvant chemotherapy improved the survival of patients with secondary localized malignant GCTB.

**Abstract:**

A malignant giant cell tumor of the bone (GCTB) is a rare primary malignant tumor classified as primary or secondary. Wide resection of the primary tumor is recommended for localized malignant GCTB, but the effect of adjuvant chemotherapy is unclear. A systematic review was performed to compare the mortality associated with wide resection with that of wide resection plus adjuvant chemotherapy for primary and secondary localized malignant GCTB. Among the 745 studies identified, 9 were included. A total of 112 cases of localized malignant GCTB were included, with 39 and 73 cases being primary and secondary malignant GCTB. In primary localized malignant GCTB, the mortality rates were 40% (6/15 patients) and 33% (8/24 patients) in the surgery plus adjuvant chemotherapy and surgery-only groups, respectively. Overall pooled odds ratio was 1.07 (95% confidence interval, 0.26–4.37; *p* = 0.92). In secondary localized malignant GCTB, the mortality rates were 30.6% (11/36 patients) and 62.2% (23/37 patients) in the surgery plus adjuvant chemotherapy and surgery-only groups, respectively. The overall pooled odds ratio was 0.31 (95% confidence interval, 0.10–0.95; *p* = 0.04). The effect of adjuvant chemotherapy remains unclear for primary localized malignant GCTB, but adjuvant chemotherapy improved the survival of patients with secondary localized malignant GCTB.

## 1. Introduction

**Figure 1:**
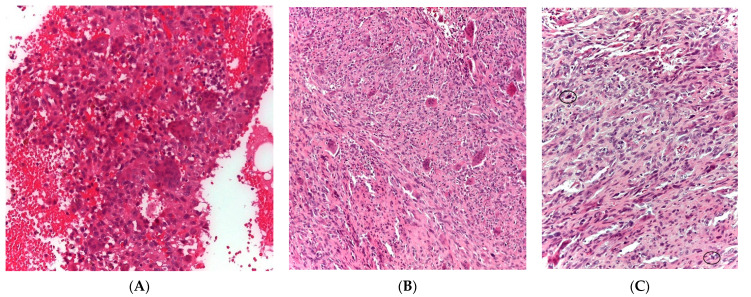
Histological specimens of primary malignant GCTB. Biopsy of a proximal humerus lesion in a 39-year-old male patient with a diagnosis of GCTB is shown. Morphologically, an oval round mononuclear cells with hemosiderotic deposits associated with multinucleated giant cells are evident (Hematoxylin and eosin [H&E], (**A**), 100× of magnification). Conversely, in the surgical specimen a transition zone between classic giant cell tumor and its relative malignant component was observed (H&E, (**B**), 100× of magnification). The malignant component of neoplasm is comprised of spindled and pleomorphic cells with an atypical mitosis (see circles in (**C**), [H&E], 200× of magnification).

**Figure 2:**
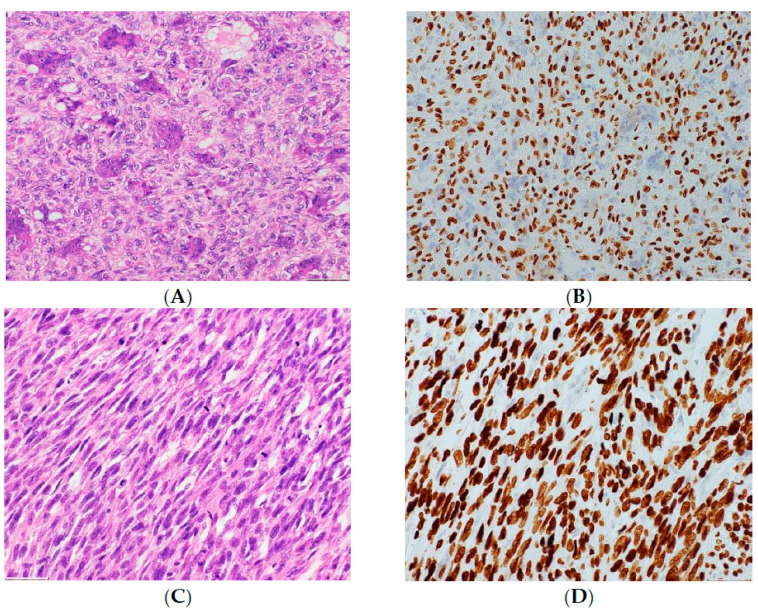
Histological specimens of secondary malignant GCTB. Histology of the initial biopsy showing multinucleated giant cells embedded in oval, round mononuclear cells with hemosiderotic deposits; the lesion presents the appearance of a classic giant cell tumor (H&E, (**A**), 200× magnification), confirmed by immuno-histochemistry to express the H3F3A protein G34W variant in mononuclear cells ((**B**), 200× magnification). Biopsy of the recurrent lesion, where a highly malignant neoplasm comprised of spindled and pleomorphic cells can be observed (H&E, (**C**), 200× magnification). Upon immunohistochemistry analysis, the H3F3A protein G34W variant was observed ((**D**), 200× of magnification).

Malignant giant cell tumors of the bone (GCTB) were first described by Stewart [1], while primary and secondary malignant GCTBs were distinguished from each other by Hutter and Dahlin et al. [2,3]. Primary malignant GCTBs are evident at the first diagnosis of GCTB and contain an area or a nodule of highly pleomorphic mononuclear cells present within an otherwise conventional GCTB (Figure 1) [4]. After treatment of primary borderline GCTB at the localized site, a few cases of malignant GCTB may be induced at the primary site. This type of malignant GCTB is called “secondary malignant GCTB at the primary localized tumor”. The pre-existing GCTB may or may not be evident (Figure 2) [4]. Malignant GCTB is a rare primary malignant tumor of the bone, with a malignant transformation rate of 2.4% for benign GCTB (secondary malignant GCTB) [5]. Benign GCTB is composed of neoplastic mononuclear stromal cells, with macrophages and multinucleated reactive giant cells (osteoclast-like) uniformly distributed [4]. The neoplastic stromal cells can originate from mesenchymal stem cells [6,7]. Benign GCTB has few somatic alterations and no driver mutations other than mutations in the H3.3 family of histone genes H3-3A or H3-3B [8]. On the other hand, malignant H3.3-mutated tumors are rich in a variety of alterations involving *TERT*; thus, telomere dysfunction is involved in the transformation from benign to malignant GCTB [8]. The mortality rate of primary malignant GCTB is reportedly lower than that of secondary malignant GCTB (16% vs. 63%) [9,10]. Although wide resection of the primary tumor and adjuvant chemotherapy are usually recommended for localized malignant GCTB [11], Anract et al. reported no difference in survival among patients who underwent surgery with adjuvant chemotherapy compared to those who received surgery alone [11]. In addition, Liu et al. reported that adjuvant chemotherapy offered no benefit on the overall survival but improved lung metastasis-free survival in patients with localized malignant GCTB [12]. Because of the rarity of malignant GCTB, only a small number of retrospective studies have been reported, and no randomized controlled trials have examined the effects of adjuvant chemotherapy for localized malignant GCTB. Therefore, the effects of adjuvant chemotherapy on localized malignant GCTBs have not yet been clarified. To investigate the efficacy of adjuvant chemotherapy for localized malignant GCTB, we performed a systematic review of studies comparing mortality in patients who underwent wide resection or wide resection plus adjuvant chemotherapy for primary and secondary localized malignant GCTB.

## 2. Materials and Methods

We followed the recommendations of the Preferred Reporting Items for Systematic Reviews and Meta-analyses (PRISMA) 2020 [13]. The protocol was registered with the UMIN Clinical Trials Registration UMIN000045042 (http://www.umin.ac.jp/ctr/index.htm (accessed on 2 August 2021)).

### 2.1. Eligibility Criteria

Only studies that reported the prognosis of localized malignant GCTB at the time of diagnosis and treated with surgery alone or surgery combined with adjuvant chemotherapy were included. Patients with distant metastases at the time of diagnosis of malignant GCTB and patients with an unclear prognosis were excluded. Patients who underwent surgery alone without adjuvant chemotherapy for the primary tumor of malignant GCTB and palliative chemotherapy for distant metastases which occurred during the course of the disease were classified into the surgery-only group. Regarding the number of deaths, only deaths due to tumors were counted. Only studies written in English or Japanese were included, and no restrictions were placed on the year of publication. Only human studies were included while in vitro and in vivo studies were excluded.

### 2.2. Literature Search and Study Selection

PubMed, Embase and Cochrane Central Register of Controlled Trials (CENTRAL) databases were used to search the literature according to a systematic search strategy on 28 July 2021 (Table S1). In addition, bibliographies of the retrieved literature were used to identify other relevant studies. Publication bias was assessed using funnel plots and the Egger’s test. Publication bias is a phenomenon in which positive results are more likely to be published than negative results when publishing a study.

### 2.3. Data Collection and Presentation

Two authors (RM and ST) independently selected the studies and extracted the data. In case of a disagreement, a consensus was reached between them or by consulting a third author. A data collection sheet was used to collate the following data: (1) basic data with authors, year of publication, journal name, type of study, follow-up period after the diagnosis of malignant GCTB, follow-up period after the diagnosis of GCTB (in the case of secondary malignant GCTB), and total number of patients with malignant GCTB; (2) number of patients treated with surgery and adjuvant chemotherapy for primary malignant GCTB and number of tumor-related deaths, and number of patients who underwent surgery alone for primary malignant GCTB and number of tumor-related deaths; (3) number of patients treated with surgery and adjuvant chemotherapy for secondary malignant GCTB and number of tumor-related deaths, and number of patients who underwent surgery alone for secondary malignant GCTB and number of tumor-related deaths; and (4) average age at diagnosis of malignant GCTB, breakdown of men and women, site of malignant GCTB, Campanacci stage of malignant GCTB [14], surgical margins for malignant GCTB, pathological diagnosis of malignant GCTB, surgery or radiotherapy for the primary lesion (benign GCTB) in the case of secondary malignant GCTB, time for malignant transformation in cases of secondary malignant GCTB, and chemotherapy regimens.

The Campanacci stage is most often used for stage classification of GCTB according to an X-ray [14]. A stage 1 the tumor has a well-marginated border consisting of a thin rim of mature bone, and the cortex is intact or slightly thinned, but not deformed [14]. A stage 2 tumor has relatively well-defined margins but no radiopaque rim; the combined cortex and rim of the reactive hone is rather thin and moderately expanded but still present [14]. Stage 3 is a tumor with fuzzy borders, suggesting rapid and possibly permeative growth; the tumor bulges into the soft tissues [14]. However, the soft-tissue mass does not follow the contour of the bone and is not limited by an apparent shell of reactive bone [14].

### 2.4. Data Summary, Synthesis and Meta-Analysis

We summarized the data collected from the selected studies (Table 1, Table 2 and Table 3). The datasets included the first author’s name, year of publication, number of patients treated with surgery combined with adjuvant chemotherapy and number of tumor-related deaths, as well as number of patients who underwent surgery only and number of tumor-related deaths. Using a random-effects model, odds ratios for comparing the rate of tumor-related deaths in the surgery-only group and the surgery plus adjuvant chemotherapy group were estimated for patients with primary and secondary malignant GCTB. The extent of heterogeneity between studies was evaluated using I^2^ statistics. All statistical analyses were performed assuming a two-sided test at a 5% level of significance using Review Manager 5.3 (The Cochrane Collaboration, Oxford, UK).

### 2.5. Assessment of Methodological Quality

Two authors (RM and ST) independently assessed the quality of the included studies. In cases of disagreement, a consensus was achieved either between them or by consulting a third author. The articles selected were independently graded for final analysis according to the Risk of Bias Assessment tool for Non-randomized Studies (RoBANS tool) to assess the quality of nonrandomized studies in the meta-analysis [22].

### 2.6. Search Results

**Figure 3:**
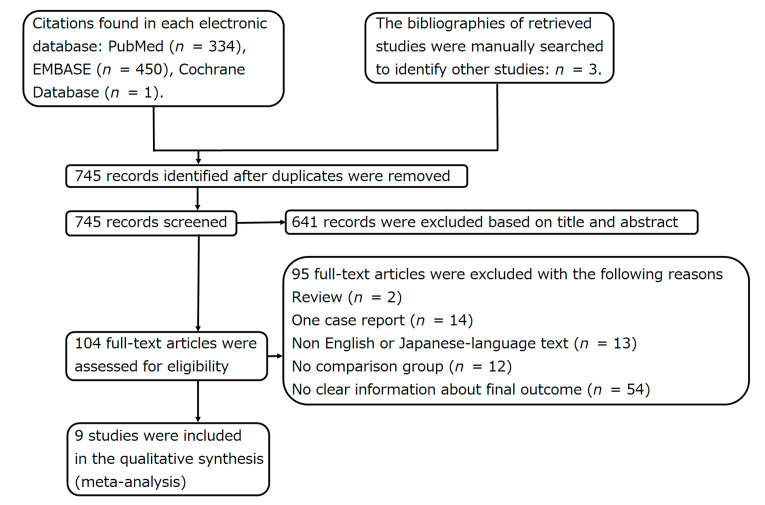
This flow chart shows the search for relevant articles.

**Figure 4:**
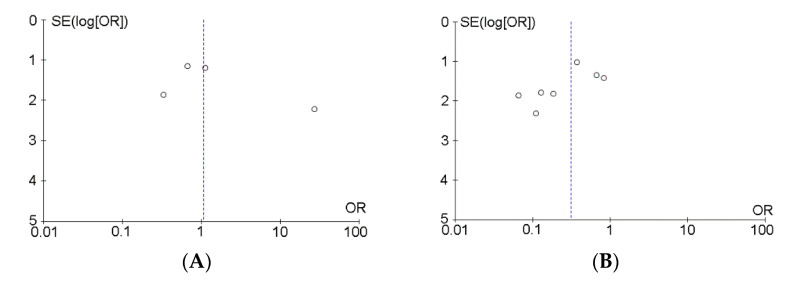
These funnel plots show detection of publication bias; (**A**) in patients with primary malignant giant cell tumor of bone; (**B**) in patients with secondary malignant giant cell tumor of bone. SE, standard error; OR, odds ratio. “o” indicates each study included the Meta-Analysis.

Among the 745 studies identified by the search, nine studies were finally included in the current study (Figure 3; Table 1, Table 2 and Table 3) [11,12,15,16,17,18,19,20,21]. None of the studies were randomized controlled trials. Additional information was obtained from the authors of two studies [19,21]. Although Egger’s test was not possible because there were only nine studies, the results of funnel plots showed that the left and right plots were asymmetrical, with the blue line as the boundary for both primary and secondary malignant GCTB, suggesting the presence of a publication bias (Figure 4A,B).

### 2.7. Demographic Data and Ratio of the Patients Who Were Treated with Surgery Combined with Adjuvant Chemotherapy and Surgery Alone

A total of 112 cases of localized malignant GCTB were included in this study, and the breakdown of primary and secondary malignant GCTB cases was 39 and 73 cases. Fifteen patients (38.5%) were treated with surgery and adjuvant chemotherapy for primary malignant GCTB, and 24 patients (61.5%) were treated with surgery alone. A total of 36 patients (49.3%) were treated with surgery and adjuvant chemotherapy for secondary malignant GCTB, and 37 patients (50.7%) were treated with surgery alone (Table 1, Table 2 and Table 3).

### 2.8. Methodological Quality of the Included Studies

The assessment of the quality of the individual studies using the RoBANS tool showed an overall moderate risk of bias. All nine included studies showed that “selection of participants” was high, “confounding variables” were high, “measurement of exposure” was low, “blinding of outcome” was low, “incomplete outcome data” was low, and “selective outcome reporting” was low.

## 3. Results

**Figure 5:**
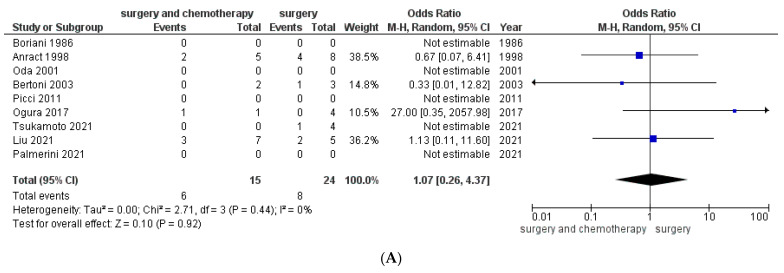

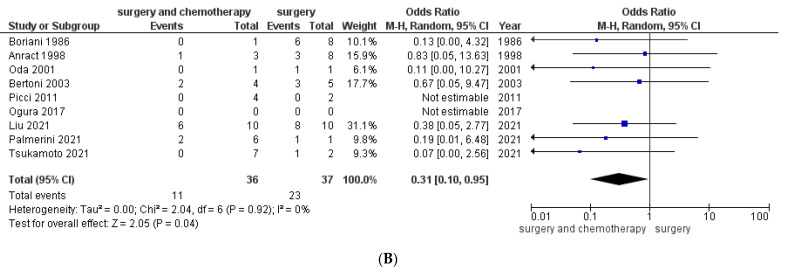
A forest plot shows the proportion of patients in the surgery plus adjuvant chemotherapy and surgery-only groups who died of disease in the various studies; (**A**) in patients with primary malignant giant cell tumor of bone; (**B**) in patients with secondary malignant giant cell tumor of bone.

In primary localized malignant GCTB, mortality was similar between the surgery plus adjuvant chemotherapy group and the surgery-only group. The mortality rates were 40% (6/15 patients) in the surgery plus adjuvant chemotherapy group and 33% (8/24 patients) in the surgery-only group. The overall pooled odds ratio was 1.07 (95% confidence interval, 0.26 to 4.37; *p* = 0.92), and the heterogeneity I^2^ was 0% (Figure 5A).

In secondary localized malignant GCTB, mortality was lower in the surgery plus adjuvant chemotherapy group than in the surgery-only group. The mortality rates were 30.6% (11/36 patients) in the surgery plus adjuvant chemotherapy group and 62.2% (23/37 patients) in the surgery only group. The overall pooled odds ratio was 0.31 (95% confidence interval, 0.10–0.95; *p* = 0.04), and the heterogeneity I^2^ was 0% (Figure 5B).

The percentage of men was 43–100% in the surgery plus adjuvant chemotherapy group, compared with 0–100% in the surgery-only group (Table 3) [11,12,15,16,17,18,19,21]. The mean age ranged from 24 to 53 years in the surgery plus adjuvant chemotherapy group, compared with 35–57 years in the surgery-only group (Table 3) [11,12,15,16,17,18,19,20,21]. The proportion of tumors located in the trunk ranged from 0% to 100% in both the surgery plus adjuvant chemotherapy group and surgery-only group (Table 3) [11,12,15,16,17,18,19,20,21]. In the surgery plus adjuvant chemotherapy group, the proportion of patients with a history of radiotherapy ranged from 0% to 100%, while in the surgery-only group, it ranged from 0% to 38% (Table 3) [11,12,15,16,17,18,21]. In the surgery plus adjuvant chemotherapy group, the proportion of inadequate surgical margins ranged from 0% to 100%, whereas in the surgery-only group, it ranged from 17% to 50% (Table 3) [12,19,21]. In the surgery plus adjuvant chemotherapy group, the proportion of osteosarcoma histology ranged from 0% to 83%, whereas in the surgery-only group, it ranged from 0% to 100% (Table 3) [11,12,15,17,18,20,21]. The mean latent period to malignant transformation ranged from 24 to 228 months in the surgery plus adjuvant chemotherapy group, compared with 36 to 264 months in the surgery-only group (Table 3) [12,15,16,17,18,20,21]. The chemotherapy regimen was reported in three studies, with the chemotherapy regimen used to treat osteosarcoma (high-dose methotrexate, cisplatin, doxorubicin, ifosfamide, and etoposide) (Table 3) [16,19,21]. Two patients with malignant GCTB included in one study were enrolled in the EUROpean Bone Over 40 Sarcoma Study (EURO-B.O.S.S.) [21]. EURO-B.O.S.S. was the first prospective study for patients 41–65 years old with high-grade bone sarcoma treated with an intensive chemotherapy regimen derived from protocols for younger patients with high-grade osteosarcoma [23]. Chemotherapy consisted of doxorubicin 60 mg/m^2^ for 24 h intravenous infusion, cisplatin 100 mg/m^2^ for 48- to 72-h intravenous infusion, ifosfamide 3 g/m^2^/day for 1- to 2-h intravenous infusions for 2 days, and methotrexate 8 g/m^2^ for 4-h intravenous infusion [23].

## 4. Discussion

The efficacy of adjuvant chemotherapy for localized malignant GCTBs remains unclear. In this study, we showed that the efficacy of adjuvant chemotherapy for primary localized malignant GCTB remains unclear, but it appears to improve survival for secondary localized malignant GCTB.

This study has several limitations. First, all included studies were retrospective and had an indication bias for adjuvant chemotherapy. Adjuvant chemotherapy was more frequently used in younger patients with a history of radiotherapy and a shorter time to malignant transformation (Table 3). Randomized controlled trials can avoid many of these biases by randomly allocating participants into groups. Because the authors identified no randomized controlled trials, well-designed cohort and observational studies with strong effects may provide reliable information. Second, since the total number of patients with primary malignant GCTB is small with only 39 patients, there is a possibility of a type 2 error. Significant results may be obtained in the future if studies on the effects of adjuvant chemotherapy in patients with localized primary malignant GCTB, which have a higher number of cases, are published. Third, based on data from the Swedish Cancer Registry from 1958 to 2011, Rockberg et al. reported that the proportion of malignant GCTBs in benign GCTBs decreased from 1.3 to 0.09 in 1982 [24]. Pathologically, it is difficult to distinguish between giant-cell-rich osteosarcomas and malignant giant cell tumors with focal areas of sarcomatous changes [25,26]. Giant-cell-rich osteosarcoma has become widely known because of the case series of Bathurst et al. published in 1986 [27]. Therefore, among the cases included in this systematic review, some of the cases may diagnose as malignant GCTB before 1982 were giant-cell-rich osteosarcomas [24]. This may affect the efficacy of adjuvant chemotherapy for localized malignant GCTBs.

The results of this study indicate that the effect of adjuvant chemotherapy remains unclear for primary localized malignant GCTB, but adjuvant chemotherapy appears to contribute to a reduction in mortality in secondary localized malignant GCTB. Primary malignant GCTB refers to tumors in which conventional GCTB and sarcoma components coexist at the time of initial diagnosis [17]. A secondary malignant GCTB is a sarcoma that develops following a benign GCTB, usually more than five years after treatment, often associated with previous radiotherapy [17]. Secondary malignant GCTB is more common than primary malignant GCTB, but both secondary and primary malignant GCTBs are very rare [4]. According to a recent systematic review, 36 cases of primary malignant GCTB were reported among 2315 patients with GCTB (incidence 1.6%), and 56 cases of secondary malignant GCTB were reported among 2315 patients with GCTB (incidence 2.4%) [5]. Nascimento et al. reported that secondary malignant GCTB has a worse prognosis than primary malignant GCTB [28]. Gong et al. reported that the mortality rate of primary malignant GCTB was 0% (0/5 patients), whereas the mortality rate of secondary malignant GCTB was 33% (4/12 patients) [29]. Mondal et al. reported that all five patients with malignant transformation after radiotherapy (secondary malignant GCTB) died within a few months [30]. In contrast, Anract et al. followed up 29 patients with malignant GCTB between 6 months and 18 years and reported they had a 5-year survival rate of 50%, and that both primary (17 patients) and secondary malignant GCTB (12 patients) had similar prognoses [11]. Liu et al. also followed up 12 patients with primary malignant GCTB and 20 patients with secondary GCTB for a mean of 4.5 years and reported a similar 5-year survival rate between primary and secondary malignant GCTB (56% vs. 40%) (*p* = 0.188) [12]. Although malignant GCTB is generally accepted to be a high-grade sarcoma [17], Domovitov et al. investigated the prognosis of 25 patients with primary malignant GCTB with a median follow-up period of 8.7 years and reported that the mortality rate of primary malignant GCTB was 16% (4 of 25 patients) [9]. According to their data, the growth rate of primary malignant GCTBs is slow [9]. This may explain why adjuvant chemotherapy is ineffective for primary malignant GCTBs.

Rock et al. reported that three patients with secondary malignant GCTB received no benefit from chemotherapy [10]. Anract et al. [11] reported improved 1-year survival in patients who underwent surgery with adjuvant chemotherapy compared to those who underwent surgery alone; however, this benefit was not observed for 5-year survival. Those authors also reported that resection specimens from three of four patients with malignant GCTB who received neoadjuvant chemotherapy showed a tumor response [11]. Liu et al. [12] found no benefit on overall survival in patients treated with adjuvant chemotherapy; however, adjuvant chemotherapy was beneficial for lung metastasis-free survival. The 5-year survival rates in the chemotherapy and non-chemotherapy groups were 57.0% and 33.3%, respectively (*p* = 0.167) [12]. Median pulmonary metastasis-free survival in patients who received chemotherapy was significantly longer than that in patients who underwent surgery alone (13 vs. 6 months; *p* = 0.002) [12]. Because of the rarity of malignant GCTB resulting in only a few reported cases, previous studies have not been able to clarify the effect of adjuvant chemotherapy on primary and secondary localized malignant GCTB. In this study, we were able to clarify the effectiveness of adjuvant chemotherapy for localized secondary malignant GCTB by collecting and analyzing previous reports. Cytotoxic chemotherapy may improve prognosis by inducing apoptosis in secondary malignant GCTB, such as in conventional high-grade osteosarcoma [31,32]. Palmerini et al. performed a systematic review of malignant GCTB and reported the frequency of primary malignant GCTB and secondary malignant GCTB, but did not investigate the effect of adjuvant chemotherapy on localized malignant GCTB [5]. The SEER database contains 250 cases of malignant GCTB but lacks information on the use of adjuvant chemotherapy and does not distinguish between primary and secondary malignant GCTB [33,34]. Therefore, the results of this systematic review may be useful for physicians treating GCTB.

## 5. Conclusions

The results of this systematic review suggest that the effect of adjuvant chemotherapy remains unclear for primary localized malignant GCTB, but adjuvant chemotherapy may improve survival in patients with secondary localized malignant GCTB. Further prospective multicenter randomized studies are needed to confirm the results of our study.

## Figures and Tables

**Table 1:** Overall study characteristics.

Study	Type of Study	Mean Follow-Up Period after Diagnosis of Malignant	Mean Follow-Up Period after Diagnosis of GCTB in the Case of Secondary Malignant GCTB (Months)	Total Number of Patients with Localized Malignant GCTB
Boriani et al. [15]	SR	30	125	9
Anract et al. [11]	SR	69	NR	24
Oda et al. [16]	SR	15	45	2
Bertoni et al. [17]	SR	58	144	14
Picci et al. [18]	SR	112	352	6
Ogura et al. [19]	MR	48	NA	5
Liu et al. [12]	SR	54	143	32
Palmerini et al. [20]	MP	Median 48	NR	7
Tsukamoto et al. [21]	MR	32	155	13

GCTB, giant cell tumor of bone; NR, not reported; NA, not applicable; SR, single institutional non-randomized retrospective study; MR, multi-institutional non-randomized retrospective study; MP, multi-institutional non-randomized prospective study.

**Table 2:** Overall study characteristics.

	In the Group of Primary Malignant GCTBs				In the Group of Secondary Malignant GCTBs			
Study	Number of Patients in Surgery Plus Adjuvant Chemotherapy Group	Number of Patients DOD in Surgery Plus Adjuvant Chemo- Therapy Group (Interval from Diagnosis of Malignant GCTB to DOD)	Number of Patients in Surgery-Only Group	Number of Patients DOD in Surgery-Only Group (Interval from Diagnosis of Malignant GCTB to DOD)	Number of Patients in Surgery Plus Adjuvant Chemotherapy Group	Number of Patients DOD in Surgery Plus Adjuvant Chemotherapy Group (Interval from Diagnosis of Malignant GCTB to DOD)	Number of Patients in Surgery-Only Group	Number of Patients DOD in Surgery-Only Group (Interval from Diagnosis of Malignant GCTB to DOD)
Boriani et al. [15]	0	0	0	0	1	0	8	6 (mean 11 months)
Anract et al. [11]	5	2 (mean 40 months)	8	4 (mean 17.5 months)	3	1 (20 months)	8	3 (mean 36 months)
Oda et al. [16]	0	0	0	0	1	0	1	1 (6 months)
Bertoni et al. [17]	2	0	3	1 (8 months)	4	2 (mean 9 months)	5	3 (mean 59 months)
Picci et al. [18]	0	0	0	0	4	0	2	0
Ogura et al. [19]	1	1 (22 months)	4	0	0	0	0	0
Liu et al. [12]	7	3	5	2	10	6	10	8
Palmerini et al. [20]	0	0	0	0	6	2 (mean 10 months)	1	1 (2 months)
Tsukamoto et al. [21]	0	0	4	1 (24 months)	7	0	2	1 (19 months)

GCTB, giant cell tumor of bone; DOD, died of disease.

**Table 3:** Overall study characteristics.

Study	Male vs. Female (Surgery + Chemo/Surgery)	Mean Age (Surgery + Chemo/Surgery)	Location of Tumor (Surgery + Chemo/Surgery)	Campanacci Stage of Malignant GCTB (Surgery + Chemo/Surgery)	Surgery of GCTB (in Case of Secondary Malignant GCTB) (Surgery + Chemo/Surgery)	Surgical Margin of Malignant GCTB (Surgery + Chemo/Surgery)	Histology (Surgery + Chemo/Surgery)	Mean Latent Period in Case of Secondary Malignant GCTB (Months) (Surgery + Chemo/Surgery)	Chemotherapy Regimen
Boriani et al. [15]	1:0/7:1	34/37	Ischium: 1/Distal radius: 1, Distal femur: 3, Proximal tibia: 3, Proximal femur: 1	NR/NR	RT alone: 1/Curettage:6, Resection: 2	NR/NR	MFH:1/MFH:3, OS:2, FS:3	30/103	NR
Anract et al. [11]	4:4/9:7	35/39	Distal femur: 2, Proximal tibia: 2, Distal radius: 1, First cuneiform: 1, Distal tibia: 1, Proximal humerus: 1/Proximal femur: 1, Distal femur: 6, Proximal tibia: 4, Distal tibia: 2, Proximal humerus: 1, Pelvis: 1, L5:1,	NR/NR	Curettage: 3/Curettage: 5, RT alone: 3	NR/NR	GCT grade III:4, OS:2, FS:2/GCT grade III:11, FS: 2, OS: 3	NR/NR	NR
Oda et al. [16]	1:0/0:1	25/42	Acetabulum: 1/Distal femur: 1	Stage 2: 1/NR	Resection: 1/Curettage: 1	NR/NR	NR/NR	24/36	CDDP + DOX
Bertoni et al. [17]	4:2/7:1	42/48	Distal femur: 3, Iliac wing: 1, Ischiopubic arch: 1, Proximal radius: 1/Proximal tibia: 3, Distal tibia: 1, Proximal femur: 1, Distal fe-mur: 2, Distal ulna: 1	NR/NR	Curettage: 3, RT alone: 1/Curet-tage: 3, Resection: 1, RT alone: 1	NR/NR	OS:5, MFH:1/OS:6, MFH:1, FS:1	90/195	NR
Picci et al. [18]	3:1/2:0	53/57	Distal femur: 1, Proximal radius: 1, Distal tibia: 1, Proximal tibia: 1/Proximal tibia: 2	NR/NR	Curettage: 4/Curettage: 2	NR/NR	OS:2, MFH:2/OS:2	228/264	NR
Ogura et al. [19]	1:0/2:2	24/49	Distal femur: 1/Distal femur: 2, Rib: 1, Proximal tibia: 1	NR/NR	NA	Inadequate: 1/Adequate: 2, Inadequate: 2	NR/NR	NA	CDDP + DOX, IFO, MTX
Liu et al. [12]	12:5/5:10	32/35	All extremity/All extremity	Stage 2: 4, Stage 3: 13/Stage 2: 4, Stage 3: 11	Curettage: 9, Resection: 1/Curettage: 8, Resection: 2	Adequate: 12, Inadequate: 5/Adequate: 11, Inadequate:	UPS or FS: 3, OS: 14/UPS or FS: 8 OS: 7	79/112	NR
Palmerini et al. [20]	NR/NR	47/55	Metatarsus: 1, Tibia: 2, Distal femur: 3/Sa- crum:1	NR/NR	NR/NR	NR/NR	UPS: 4, OS: 2/UPS: 1	82/79	NR
Tsukamoto et al. [21]	3:4/6:0	31/47	Sacrum: 2, Extremity: 5/Extremity: 6	NR/NR	RT alone: 1, RT + Curettage:1, Curettage: 5/Curettage: 1, Resection: 1	Adequate: 6, Carbon ion: 1/Adequate: 5, Inadequate: 1	OS: 3, UPS: 4/OS: 1, UPS: 5	172/64	CDDP, DOX, IFO, VP-16, MTX

GCTB, giant cell tumor of bone; NR, not reported; NA, not applicable; RT, radiotherapy; OS, osteosarcoma; MFH, malignant fibrous histiocytoma; UPS, undifferentiated pleomorphic sarcoma; FS, fibrosarcoma; MTX, methotrexate; CDDP, cisplatin; IFO, ifosfamide; DOX, doxorubicin; VP-16, etoposide.

## Supplementary Materials

The following are available online at https://www.mdpi.com/article/10.3390/cancers13215410/s1, Table S1: Search strategy.
