# Supplementary material for: Effect of Adjuvant Chemotherapy on Localized Malignant Giant Cell Tumor of Bone: A Systematic Review
Source: Cancers (Basel). 2021 Oct 28;13(21):5410. doi: 10.3390/cancers13215410 (PMC8582404; doi:10.3390/cancers13215410)
Supplement: Supplementary file 1 [file cancers-13-05410-s001.zip › cancers-1358398-supplementary.pdf]

cancers

MDPI

Supplementary Materials: Effect of Adjuvant Chemotherapy on Localized Malignant

Rokuro Morii, Shinji Tsukamoto, Alberto Righi, Kanya Honoki, Yuu Tanaka, Akira Kido, Hiromasa Fujii, Andreas F. Mavrogenis, Yasuhito Tanaka and Costantino Errani

Table S1. Search strategy.

**Ovid Medline**	**Search Strategy**	**2021/7/28**
Search history sorted by search number ascending		
# ▲	Searched for	Results
1	(malignan* adj3 "giant cell" adj1 tumo*).mp.	312
2	(malignan* adj3 GCTB*).mp.	36
3	1 or 2	334
Embase		2021/7/28
Set#	Searched for	Results
S1	(TI,AB((malignan* N/3 "giant cell" P/0 tumo*))) OR (TI,AB(malignan* N/3 GCTB*))	547
S2	S1 and (dstat.exact("Embase" OR "Article in Press" OR "In Process"))	450
Duplicates are removed from the search and from the result count.		
Central		2021/7/28
ID	Search	Hits
#1	((malignan* NEAR/3 ("giant cell" NEXT tumo*)) OR (malignan* NEAR/3 GCTB*)):ti,ab,kw	2
#2	pubmed:an	719229
#3	#1 not #2	1
